# Incorporating and Emphasizing Sexual Health in Medical Curriculum of Nepal: Need of the Hour

**DOI:** 10.31729/jnma.7454

**Published:** 2022-06-30

**Authors:** Reet Poudel, Vikash Paudel

**Affiliations:** 1 Department of Psychiatry, Nepalgunj Medical College, Kohalpur, Nepal; 2 Department of Dermatology and Venereology, National Medical College and Teaching Hospital, Birgunj, Nepal

**Keywords:** *medical education*, *Nepal*, *sexual health*

## Abstract

Sexual health requires a positive and respectful approach to sexuality and sexual relationships. It is one of the most neglected parts of an individual's health. Sexual health is not adequately covered in the medical education curriculum of Nepal. There is a lack of clinicians practising sexual medicine, which provides a fertile field for quacks in this arena. Sexual health needs to be included and incorporated into medical education. The policymakers and stakeholders need to address this need in sexual health urgently and effeciently. Comprehensive sexual education should be included for children, adolescents and young adults.

## INTRODUCTION

The World Health Organization (WHO) has defined sexual health as a “state of physical, emotional, mental and social well-being in relation to sexuality; it is not merely the absence of disease, dysfunction or infirmity".^1^ Sexual health requires a positive and respectful approach to sexuality and sexual relationships, as well as the possibility of having pleasurable and safe sexual experiences, free of coercion, discrimination and violence.^1^ Sexuality has always been a subject of importance to the medical community and a focus of interest to humankind. It is determined by anatomy, physiology, culture in which a person lives, relationships with others, and developmental experiences throughout life.^2^

## CURRENT SCENARIO

Despite the fact that sex and sexuality-related issues are commonly encountered especially in medical practice, sexual health has been one of the most neglected part of individual health. Most of the topics covered in medical education on sexual health are related to reproductive health and sexually transmitted infections like genital ulcer diseases, diseases associated with discharge from genitalia, Human Immunodeficiency Virus Infection/Acquired Immune Deficiency Syndrome (HIV/AIDS), etc. while other issues like gender identity, comprehensive sexuality education, sexual dysfunctions and Lesbian, Gay, Bisexual, and Transgender (LGBTQ + ) issues are inadequate or absent.

Literature from the western world has shown sexual dysfunctions are highly prevalent in both sexes, more in women (25-63%) than men (10-52%).^3-6^ An epidemiological study from India has found that one in five males and one in seven females have one or more sexual disorders.^7^ The common problems related to sexual health are premature ejaculation, erectile dysfunction, delayed ejaculation, nocturnal emission, Dhat syndrome, myths related to sex and sexuality, performance anxiety, guilt about masturbation, hypo-active sexual desire, anorgasmia, vaginismus, dyspareunia, sexual aversion, and infections and tumours of the reproductive system.^3,7,8^

Though some issues related to sexually transmitted diseases and the reproductive tract are dealt by venereologists, gynecologists and some urologists, these issues are not dealt adequately in medical school as there is a lack of clinicians practising sexual medicine in Nepal. Discussing about sex and sexual disorders is stigmatized and is considered a taboo. Besides, patients with sexual problems need privacy and are hesitant for checkups and investigations as they feel embarrassed.

These problems might disseminate to other areas and can later cause complications, disrupting the quality of life.^9^ This can lead to mental distress, and a feeling of guilt among patients, leading them to refrain from visiting doctors, and rather seek help from chemists, friends, the internet and so on.

## A NEGLECTED ISSUE

In the Indian subcontinent, knowledge about sexual health is obtained from friends, magazines, internet, radio, social media and pornographic movies. A primary care physician especially in a rural setting is expected to address the common sexual health problems, but the medical graduates lack this basic knowledge because they were never taught about these issues in their medical school.^10^ Most sexual and reproductive health services in Nepal are provided through private and public health centres. These include local pharmacists, public health practitioners, doctors, nurses, and community health workers. Young people obtain sexual health services when they visit health centres, hospitals, or clinics. However, many such programmes are poorly implemented.^11^ Even in an urban setting many doctors hesitate or are uncomfortable inquiring about their patient's sexual health.

If patients complain about a sexual health issue, doctors are unaware of the ways to approach these issues or to take a proper sexual history. Some even deem the sexual issues of the patient as unimportant and focus on other health issues. Unfortunately, this topic is often ignored in the syllabus and training of both undergraduate and postgraduate medicine.

Due to this shortage of trained medical professionals practising sexual medicine, patients especially males are exploited both emotionally and financially by quacks or untrained professionals.^12^ Sexual health related problems have always been a breeding ground for these untrained professionals. Posters advertising solutions to every sexual health related problem are strategically positioned on fuse boxes and dark walls which are impossible to miss. These posters attract males with sexual dysfunctions and lead them to the misery of quacks. Since patients don't know how to distinguish them from bonafide practitioners, they run to them. Instead of their problems being solved, it makes the patient both mentally and economically drained.

In our part of the world, most of the sexual health problems are dealt by a variety of specialists including physicians, psychiatrists, dermatologists and gynaecologists.^13,14^ Even among them, few may not prefer to practice sexual medicine as sex is still considered to be a taboo in this part of the world.^10^ For those clinicians who are interested to study human sexuality and sexual health, hardly any specific courses on sexual medicine exist. Many clinicians practising in this field 'double' as sexologists or sexual health experts while pursuing their original discipline i.e., while practising their original field, they also work as sexologists. Many homeopaths, ayurveda doctors and faith healers also dabble in the field.

## HOPES FOR FUTURE

In a recent development, in September 2021; the Madras High Court of India directed the National Medical Commission (NMC) to make amendments to the medical syllabus under the Central Board of Medical Education (CBME) curriculum and remove any derogatory or unscientific remarks about the LGBTQ + community. Additionally, Madras High Court ordered all medical educational institutions providing Under/Post Graduate degrees to teach gender-related issues in a manner that is not derogatory, discriminatory, or insulting to the queer community. All medical institutions, colleges, and universities have been asked to refrain from annexing books as 'recommended' books by the university which provide unscientific, derogatory, and discriminatory information about virginity and the LGBTQ + community.^14^ As most of the medical textbooks used in Nepal are mostly from Indian writers/publishers; this change is expected to reflect positively on medical education/curriculum in Nepal to some extent.

## WAY FORWARD

The current medical education and syllabus regarding sexual health need to be changed urgently. Sexual health needs to be included and incorporated in both the undergraduate and postgraduate syllabus of medical education. Approaching a patient with various sexual health issues, taking a proper sexual history, counselling, accurate diagnosis and management options including timely referral need to be taught to our fellow doctors. A specific post-graduation course of Doctor of Medicine (MD), fellowship, or at least a short-accredited course in sexual health or sexual medicine can be introduced by various universities in Nepal so that competent doctors can be produced to identify and reduce the burden of sexual disorders; and also to cater the sexual health needs of the general population. Comprehensive sexual education should be included for school children. Various approaches including the use of mass media, the internet and the establishment of youth-friendly service centres in convenient places can be initiated to implement sexuality education and enable adolescents and young adults to seek for sexual health without social stigma attached to it.
